# High Variability of Fabry Disease Manifestations in an Extended Italian Family

**DOI:** 10.1155/2015/504784

**Published:** 2015-04-22

**Authors:** Giuseppe Cammarata, Pasquale Fatuzzo, Margherita Stefania Rodolico, Paolo Colomba, Luigi Sicurella, Francesco Iemolo, Carmela Zizzo, Riccardo Alessandro, Caterina Bartolotta, Giovanni Duro, Ines Monte

**Affiliations:** ^1^Institute of Biomedicine and Molecular Immunology (IBIM), National Research Council, 90146 Palermo, Italy; ^2^Internal Medicine, Nephrology and Dialysis Unit, Department of Medical and Pediatric Sciences, University of Catania, 95100 Catania, Italy; ^3^Institute of Neurological Sciences (ISN), National Research Council, 95126 Catania, Italy; ^4^Complex Operative Unit of Neurology, S. Antonio Abate Hospital, 91016 Trapani, Italy; ^5^Department of Neurology, “R. Guzzardi” Hospital, ASP Ragusa, Vittoria, 97019 Ragusa, Italy; ^6^Department of Biopathology and Medical and Forensic Biotechnology, Section of Biology and Genetics, University of Palermo, 90133 Palermo, Italy; ^7^Cardio-Thorax-Vascular and Transplant Department, University of Catania, 95125 Catania, Italy

## Abstract

Fabry disease (FD) is an inherited metabolic disorder caused by partial or full inactivation of the lysosomal hydrolase *α*-galactosidase A (*α*-GAL). The impairment of *α*-GAL results in the accumulation of undegraded glycosphingolipids in lysosomes and subsequent cell and microvascular dysfunctions. This study reports the clinical, biochemical, and molecular characterization of 15 members of the same family. Eight members showed the exonic mutation M51I in the GLA gene, a disease-causing mutation associated with the atypical phenotype. The clinical history of this family highlights a wide phenotypic variability, in terms of involved organs and severity. The phenotypic variability of two male patients is not related to differences in *α*-GAL enzymatic activity: though both have no enzymatic activity, the youngest shows severe symptoms, while the eldest is asymptomatic. It is noticeable that for two female patients with the M51I mutation the initial clinical diagnosis was different from FD. One of them was diagnosed with Familial Mediterranean Fever, the other with Multiple Sclerosis. Overall, this study confirms that the extreme variability of the clinical manifestations of FD is not entirely attributable to different mutations in the GLA gene and emphasizes the need to consider other factors or mechanisms involved in the pathogenesis of Fabry Disease.

## 1. Background

Anderson-Fabry disease is a metabolic lysosomal storage disorder caused by the functional deficit of the enzyme *α*-galactosidase A (*α*-GAL A) [1]. This deficit alters the metabolism of some glycosphingolipids, mainly globotriaosylceramide (Gb3), that accumulate in lysosomes of many cell types [2]. FD is an X-linked lysosomal enzymopathy caused by mutations in the GLA gene coding for *α*-GAL A located on the long arm of the X Chromosome (Xq22.1) [3]. To date, more than 1000 mutations were described in the GLA gene's exons and introns and discrimination between pathological and neutral mutations is difficult (http://fabry-database.org/, June 2013) [4, 5]. Gb3 and other neutral glycolipids gradually accumulate in endothelial cells, smooth muscle cells in blood vessels, renal epithelial cells, pericytes, myocardial cells, neurons of the spinal cord, and neurons of the dorsal root ganglia; this leads progressively to cellular dysfunction, necrosis, apoptosis, inflammation, fibrosis, and poor target-organs perfusion. Clinical manifestations are more severe in male hemizygous subjects than in female heterozygotes subjects, often asymptomatic, according to* Mary Lyon*'s hypothesis of random inactivation of the X Chromosome [6]. Recently it was found that also women can show severe manifestations of FD with irreversible organ damage, thus excluding the theory that women are exclusively carriers of the disease. For this reason, an accurate follow-up is required for female patients, independently from enzymatic activity levels.

The clinical manifestations of the disease appear in childhood; harmful, renal, cardiac, and cerebrovascular complications usually arise in the adulthood. The selective damage of tubular epithelial cells and glomerular epithelial cells in FD patients often causes chronic kidney disease that progresses to end-stage renal disease (ESRD) with age [7]. The enzyme replacement therapy (ERT) is the current treatment available for Fabry patients that reduces Gb3 levels and prevents further accumulation [8]. Although the ERT blocks the progression of the disease in most of the cases, it is not able to limit some specific symptoms and to eradicate the disease [9–11]. A reliable and early diagnosis of Fabry disease is still hard to perform. Retrospective studies found a significant delay of the diagnosis of Fabry disease in about 40% of males and 70% of females [12]. In particular, the time elapsing between the onset of first signs and symptoms and the correct diagnosis is 13 years for male patients and 17 years for female patients [13]. Probably the difficulties in diagnosis make the impact of the disease hard to assess; the incidence in the general population is estimated at 1 : 40,000, but recent initiatives of neonatal screening found an incidence of 1 : 3,100 born in Italy [14] and 1 : 1,500 in newborns in Taiwan [15]. FD is underestimated mainly because its clinical manifestations can be confused with those of other systemic diseases. For this reason, FD is often treated as single organ pathology. In literature there are cases in which the clinical indicators of FD overlap those of some rheumatic disorders, such as Familial Mediterranean Fever (FMF). In effect, these two diseases share not only the first symptoms, but also the clinical manifestations; the first signs appear during childhood, with recurrent episodes of fever, abdominal and joint pain, gastrointestinal disorders, and kidney damage. FD often involves also the central nervous system causing micro- and macroangiopathy in the brain. Because of these features and the frequent presence of lesions in MRI scans, FD is often misdiagnosed as Multiple Sclerosis [16]. In this paper, we report the study of an interesting family group and our results confirm several critical issues related to FD and probably evoke new questions.

## 2. Patients and Methods

### 2.1. Patients

Peripheral blood samples were collected, using EDTA as an anticoagulant, for genetic analysis and detection of *α*-galactosidase A activity.

### 2.2. DNA Isolation

DNA samples were isolated from whole blood by column extraction (GenElute Blood Genomic DNA Kit, Miniprep, Sigma-Aldrich, USA), and their concentrations were determined using a spectrophotometer.

### 2.3. HRM Analysis and DNA Sequencing

A presequencing screening was performed on DNA samples by High Resolution Melting (HRM) analysis for the study of the exons of the GLA gene and their flanking regions; the Light Cycler 480 system (Roche Applied Science, Germany) was used. PCR products presenting melting curves different in position or shape from those of the wild type control were sequenced to identify the suspected mutations. Purified PCR products were sequenced using the automated DNA sequencer at BMR Genomics.

### 2.4. *α*-Galactosidase A Activity Assay

The activity of *α*-galactosidase A was determined by the Dried Blood Filter Paper test described by Chamoles et al., with minor modifications [17].

## 3. Results and Discussion

**Figure 1:**
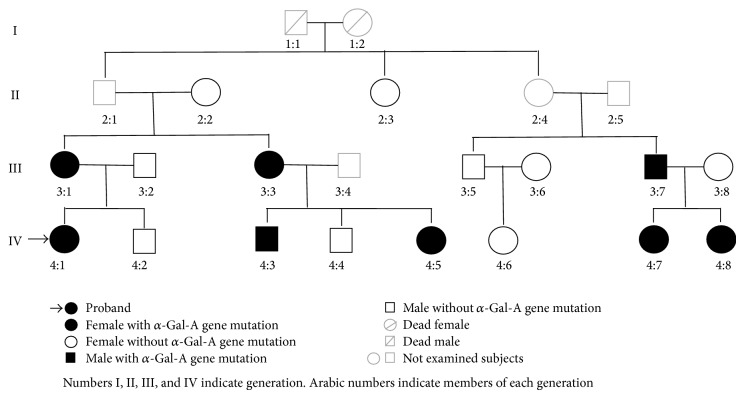
The family tree/pedigree.

**Figure 2:**
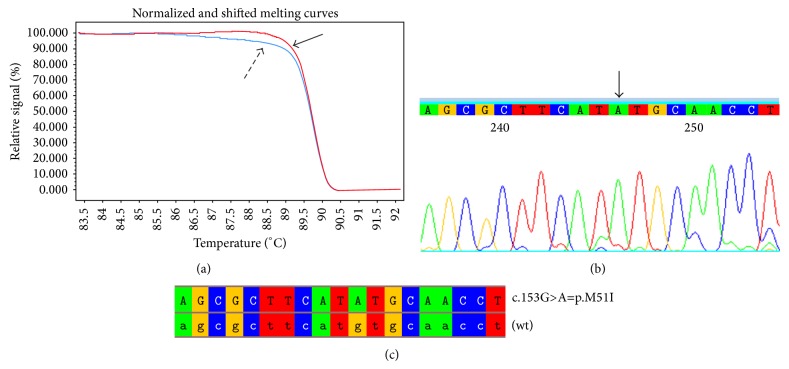
Mutation p.M51I: (a) HRM analysis of exon 1 of the GLA gene in male patients (curves indicated by the dashed arrow) and in a healthy control (curves indicated by the solid arrow); (b) portion of the electropherogram of exon 1 of the GLA gene in male patients in which p.M51I mutation is indicated by the arrow; (c) portion of the sequence of exon 1 of the GLA gene in male patients aligned with the corresponding sequence of a healthy control (wt).

This study reports the clinical, biochemical, and molecular characterization of 15 members of the same family. The pedigrees of the family are shown in Figure 1 and relevant enzymatic and molecular data are given in Table 1. The molecular analysis of the GLA gene revealed the known M51I mutation in 8 patients aged from 22 to 58 years (mean 34.6 ± 15.96) [14] as in Figure 2. A young 22-year-old female patient (case  4:1) came at our attention because during her childhood she had suffered from recurrent fever of unknown origin, burning pain in hands and feet, and gastrointestinal disturbances. She underwent several instrumental and genetic tests that led to the clinical diagnosis of FMF. This diagnostic hypothesis was not supported by the genetic analysis of the MEFV gene, showing a single heterozygous mutation (A744S). Therefore, Fabry disease was considered for diagnosis. The genetic analysis of the GLA gene was performed and the M51I mutation was identified. Ten years after the appearance of the first clinical manifestations, the definitive diagnosis was made: Fabry disease. The patient harboring a pathogenic mutation showed an enzymatic activity of 2.6 nmol/h/spot, below the normal values. Genetic and biochemical tests were extended to proband family members. These analyses showed that the proband inherited the M51I mutation from her mother (case  3:1). She is a 56-year-old woman with the enzymatic activity slightly above normal values and with symptoms not clearly related to FD. The proband's father (case  3:2) showed neither mutations nor symptoms and normal enzymatic activity (3.5 nmol/h/spot). The maternal aunt of the proband (case  3:3) was found to carry the same mutation and her enzymatic activity was 2.9 nmol/h/spot, below normal range. This 53-year-old woman, unlike her sister (case  3:1), showed cardiac involvement with dyspnea and arrhythmias. More severe symptoms affected the children of case  3:3, who are actually cousins of the proband. Among them, a young woman aged 21 (case  4:5) with the M51I mutation and low enzymatic activity (2 nmol/h/spot) showed angiokeratomas and moderate proteinuria; case  4:3 is a young man aged 28 with the M51I mutation, no enzymatic activity, and showing a serious cerebrovascular involvement. Another member of this family with no enzymatic activity is case  3:7 that is the cousin of the proband's mother (case  3:1). Although this 58-year-old man carries the same mutation found in his relatives, he is completely asymptomatic. This man's daughters also inherited the M51I mutation; the 22-year-old daughter (case  4:7) had the *α*-galactosidase A activity below normal range (2.4 nmol/h/spot); the other daughter aged 25 (case  4:8) had no enzymatic activity, a rare condition for female subjects, and was also diagnosed as affected by Multiple Sclerosis based on the presence of lesions of the corpus callosum on MRI. Noteworthy is that the brother (case  3:5) of case  3:7 had lower than normal enzymatic activity (2.3 nmol/h/spot), though he had neither genetic alteration in the GLA gene nor symptoms referable to Fabry disease.

## 4. Conclusions

Fabry disease is a lysosomal storage disorder with the glycosphingolipid metabolism deeply compromised.

The defect of *α*-galactosidase A leads to the progressive accumulation of globotriaosylceramide in parenchyma cells of various organs and in endothelial cells. This pathology is believed to be rare, but it should be considered uncommon, little known, according to results from literature [13]. Fabry Disease is still hard to diagnose because of its features and because it has clinical manifestations overlapping to other pathologies and subsequently a wide range of differential diagnoses that involve several clinical specialties [18]. In particular, subjects affected by the atypical form of the disease are harder to diagnose than subjects with the classic phenotype. In this study we report the clinical, biochemical, and molecular study of 15 members of the same family. In addition to the proband, seven subjects showed alterations in the GLA gene. This aspect highlights the importance of pedigree analysis in families with FD for identifying other possibly affected relatives [19]. Precisely the genetic analysis revealed the M51I mutation, which is considered pathogenic and related to the atypical variants of the disease [14, 20]. The M51I mutation is a G to A transition at the codon 51 that causes the substitution between two nonpolar amino acids: Methionine and Isoleucine. Molecular models of this mutated protein showed that this mutation does neither alter the active site of *α*-galactosidase A nor interfere with the enzyme catalytic activity, but it could alter the enzyme stability [21]. The analysis of this family confirmed that for this disorder there is little genotype-phenotype correlation, even at intrafamiliar level, and that it can cause a constellation of symptoms, often overlapping to different pathologies. These critical features of Fabry disease frequently cause misdiagnosis, delay in the correct diagnosis, and difficulties in prognosis. The current pathogenic theory of FD is based on the fact that the GLA gene is the only one associated with this disorder and that the defect in *α*-galactosidase A is responsible for the accumulation of GB3 in lysosomes and for the subsequent cell damage and clinical symptomatology. Symptoms variability could be related to the residual enzyme activity in nonclassic FD patients and to the progression of FD, whose clinical manifestations get worse over time. Nevertheless, this theory is contradicted by some cases found in this and other studies [22, 23]. Particular is the case of the two male members of this family hemizygous for the M51I mutation. Though both have no enzymatic activity, they show different clinical manifestations; the youngest (28 years old) has more severe symptoms than the eldest (58 years old), who is asymptomatic. Therefore, considering the current pathogenic theory, it is hard to explain the absence of symptoms in a patient with no enzymatic activity and his survival over the fifth decade of life. The penetrance of different GLA mutations could be influenced by the interindividual variability of other genetic and epigenetic factors or by differences in environmental conditions. It is still not clear if the extreme phenotypic disease variability and the involvement of different target organs could be explained with these processes individually or taken together [24]. Further investigations are required to understand the reasons for this variability to improve the accuracy of the prognosis and diagnosis of FD.

## Figures and Tables

**Table 1:** GLA genetic diagram of family studied.

Patient number	Kinship	Gender/age	GLA mutation	Enzyme activity (nmol/h/spot)
2:2	Grand mother	F/84	—	—
2:3	Sister of 2:2	F/77	—	3.1
3:1	Mother	F/56	M51I	3.3
3:2	Father	M/53	—	3.5
3:3	Aunt	F/53	M51I	2.9
3:5	Cousin of 3:1	M/64	—	2.3
3:7	Cousin of 3:1	M/58	M51I	0.3
4:1	Proband	F/22	M51I	2.6
4:2	Brother	M/27	—	5.5
4:3	Cousin	M/28	M51I	0
4:4	Cousin	M/25	—	—
4:5	Cousin	F/21	M51I	2
4:6	Daughter of 3:5	F/24	—	5.4
4:7	Daughter of 3:7	F/22	M51I	2.4
4:8	Daughter of 3:7	F/25	M51I	0

—, no mutation; F, female; M, male. Normal values of *α*-Gal-A activity assayed in whole blood are >3 nmol/h/spot.
